# Vascular Endothelial Growth Factor Inhibits Phagocytosis of Apoptotic Cells by Airway Epithelial Cells

**DOI:** 10.1155/2020/5287131

**Published:** 2020-08-17

**Authors:** Mimi Mu, Peiyu Gao, Jing He, Xiangnan Tao, Chuanwang Song

**Affiliations:** ^1^Department of Immunology, School of Laboratory Medicine, Bengbu Medical College, Anhui 233030, China; ^2^Anhui Provincial Key Laboratory of Infection and Immunity, Bengbu Medical College, Bengbu, Anhui 233030, China; ^3^Anhui Province Key Laboratory of Immunology in Chronic Diseases, Bengbu Medical College, Bengbu, Anhui 233030, China; ^4^Clinical Laboratory, The Second Affiliated Hospital of Bengbu Medical College, Anhui 233004, China

## Abstract

Professional phagocytes such as dendritic cells and macrophages can ingest particles larger than 0.5 *μ*m in diameter. Epithelial cells are nonprofessional phagocytes that cannot ingest pathogenic microorganisms, but they can ingest apoptotic cells. Inhibition of the engulfment of apoptotic cells by the airway epithelium can cause severe airway inflammation. Vascular endothelial growth factor (VEGF) is an angiogenesis-promoting factor that can mediate allergic airway inflammation and can promote airway epithelial cells (AECs) proliferation, but it is not clear whether it affects the engulfment of apoptotic cells by AECs. In the present study, VEGF inhibited engulfment of apoptotic cells by AECs via binding to VEGF receptor(R)2. This inhibitory effect of VEGF was not influenced by masking of phosphatidylserine (PS) on the surface of apoptotic cells and was partially mediated by the PI3K-Akt signaling pathway. VEGF inhibition of phagocytosis involved polymerization of actin and downregulation of the expression of the phagocytic-associated protein Beclin-1 in AECs. Since engulfment of apoptotic cells by AECs is an important mechanism for airway inflammation regression, VEGF inhibition of the engulfment of apoptotic cells by airway epithelial cells may be important for mediating allergic airway inflammation.

## 1. Introduction

Phagocytic cells can engulf particles larger than 0.5 *μ*m in diameter [1]. Specialized or professional phagocytes like dendritic cells (DCs) and macrophages play an important role in the resistance to infection by pathogenic microbes and maintaining homeostasis. Nonprofessional phagocytes including epithelial cells and fibroblasts cannot phagocytize pathogenic microorganisms but can ingest apoptotic cells, a process known as efferocytosis [2, 3]. Apoptosis, or programmed cell death, is a key process in tissue homeostasis, and phagocytosis of apoptotic cells by the tissue epithelium protects against inflammatory or immunogenic responses to the dying cells [4]. A recent study by Juncadella et al. found that phagocytosis of apoptotic airway cells depended on Ras-related C3 botulinum toxin substrate 1 (Rac1), which is a GTPase, and that defective phagocytosis contributed to allergic airway inflammation [5]. Asthma is a chronic airway inflammation involving a number of different cells and diverse cellular components [6]. Previous studies described VEGF-mediated allergic airway inflammation in asthmatic mice [7, 8]. The mechanism is not clear, but it is known that the angiogenesis-promoting factor VEGF can promote the survival and proliferation of airway epithelial cells (AECs) [9]. It is not known whether VEGF can promote phagocytosis by AECs.

Recent studies have described VEGF involvement in macrophage phagocytosis. VEGF expression was negatively correlated with the phagocytic function of macrophages in a study finding that treatment with T11 target structure membrane glycoprotein promoted phagocytosis and reduced VEGF expression [10]. The binding of lectin agglutinin by immature DCs was found to inhibit phagocytosis and increase intracellular VEGF [11]. This study investigated the effect of VEGF on efferocytosis by AECs. VEGF inhibited epithelial cell efferocytosis via VEGFR2, was not affected by the masking of PS on the surface of apoptotic cells, and was partially mediated by the PI3K-Akt pathway.

## 2. Materials and Methods

### 2.1. Isolation and Culture of AECs

Primary AECs were isolated from BALB/c mice as previously described [5]. Briefly, mice were intubated and leukocytes were removed by bronchoalveolar lavage with phosphate-buffered saline (PBS), and the lungs were then lavaged with PBS via the pulmonary artery until they appeared white. Lung tissue was removed and digested at 37°C for 30 min with 0.25% trypsin, 100 *μ*g/ml DNase, and 40 *μ*g/ml collagenase. The resulting liquid was passed through 200 and 400 mesh filters; the filtrate was washed twice with PBS containing 1% fetal bovine serum (FBS), and the red blood cells were removed by centrifugation. After resuspending and washing the cell suspension, CD45-positive cells were removed by magnetic bead sorting. CD45-negative cells were resuspended in Dulbecco's modified Eagle medium containing 10% FBS, inoculated into 6-well plates, and cultured at 37°C for 2 h. Nonadherent cells were collected and seeded into 6-well plates precoated with collagen and maintained in a 37°C incubator until use.

### 2.2. Induction and Assay of Apoptosis

AECs were seeded into 6-well plates at 1 × 10^6^/well and stimulated with 50 *μ*M of curcumin for 72 h. The cells were washed twice with PBS containing 1% FBS, and then gently resuspended in 195 *μ*l Annexin V-fluorescein isothiocyanate (FITC) binding solution followed by incubation with 5 *μ*l of Annexin V-FITC for 20 min at room temperature. Apoptotic cells were assayed with a FACSCalibur flow cytometer.

### 2.3. Assay of Phagocytosis

AECs were seeded in 12-well plates at 5 × 10^4^/well, stimulated with 1 ng/ml, 10 ng/ml, 100 ng/ml, 300 ng/ml, 500 ng/ml, or 1000 ng/ml VEGF for 6 h, before adding 1 × 10^5^ fluorescein isothiocyanate (FITC)-labeled curcumin-induced apoptotic cells. After 4 h, the epithelial cells were washed twice with PBS before assay of phagocytosis by FITC fluorescence using a FACSCalibur flow cytometer. To assay the blocking of phagocytosis, epithelial cell cultures were treated with anti-VEGFR2 antibodies, Wortmannin, or cytochalasin D for 2 h before stimulation with 500 ng/ml VEGF.

### 2.4. Assay of PS on the Surface of Apoptotic Cells

Cells were washed with PBS, suspended in Annexin V-FITC binding solution, and incubated at room temperature with Annexin V-FITC staining solution for 20 min. Surface PS was assayed with a FACSCalibur flow cytometer.

### 2.5. Assay of VEGFR2 on the Surface of AECs

Cells were seeded in 6-well plates, incubated for 6 h, washed twice with PBS containing 1% FBS, and then incubated with FITC-labeled VEGFR2 antibody for 30 min on ice. The cells were then washed twice with PBS and fixed with 4% paraformaldehyde. Cell surface expression of VEGFR2 was assayed by flow cytometry.

### 2.6. Western Blot Assay

Cellular proteins were extracted with NP40 cell lysis buffer, and the protein concentration was determined with a bicinchoninic acid assay. The extracted proteins (30 *μ*g per channel) were separated on 10% sodium dodecyl sulfate–polyacrylamide gels by electrophoresis and then transferred to nitrocellulose membranes. Membranes were blocked with 5% BSA for 2 h at room temperature and incubated overnight at 4°C with anti-VEGFR2, anti-p-Akt, anti-Beclin1, anti-*β*-actin, and anti-GAPDH antibodies. The membranes were then incubated with horseradish peroxidase-labeled goat antirabbit secondary antibody for 2 h at room temperature. The blots were visualized with a Beyo Electrochemiluminescence (ECL) Plus kit, and optical density analysis was performed with Image J software.

### 2.7. Statistical Analysis

Data were reported as means ± standard deviation. Statistical analysis was performed using SPSS 10.0 software. Analysis of variance was used for group comparisons. *P* values <0.05 was considered statistically significant.

## 3. Results

### 3.1. VEGF Inhibits Efferocytosis by AEC

**Figure 1:**
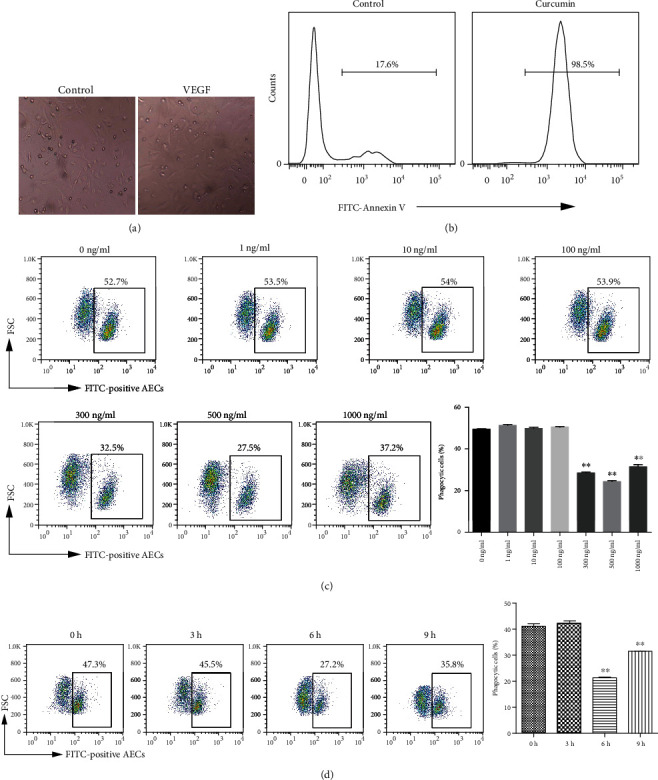
VEGF inhibits the engulfment of apoptotic cells by AECs. (a) Isolated primary AECs were inoculated into 6-well plates (5 × 10^4^ cells/well) and treated with 500 ng/ml VEGF for 6 h. (original magnification ×400). (b) Epithelial cells were inoculated into 6-well plates (1 × 10^6^ cells/well) and treated with 50 *μ*M curcumin for 72 h. Cell surface PS expression was assayed by flow cytometry. (c) Epithelial cells were inoculated into 12-well plates (5 × 10^4^ cells/well) and treated with 1, 10, 100, 300, 500, or 1000 ng/ml VEGF for 6 h before incubation with 10^5^ FITC-labeled curcumin-induced apoptotic cells for 4 h. After washing with PBS, phagocytosis by epithelial cells was assayed detection of fluorescence by flow cytometry. ^∗∗^*P* < 0.01 compared with 0 ng/ml VEGF. (d) Epithelial were seeded in 12-well plates (5 × 10^4^ cells/well) and treated 500 ng/ml with VEGF for 3, 6, or 9 h before incubating for 4 h with FITC-labeled curcumin-induced apoptotic cells. Engulfment of apoptotic cells was assayed by flow cytometry. ^∗∗^*P* < 0.01 compared with 0 h.

The morphology of cultured primary AECs was not changed by 500 ng/ml VEGF (Figure 1(a)). After 72 h incubation with 50 *μ*M curcumin, >98% of the AECs became Annexin V-binding PS-positive apoptotic cells (Figure 1(b)). Airway epithelial cell cultures were stimulated with VEGF for 6 h and then cocultured with FITC-labeled apoptotic cells. As shown in (Figure 1(c)), 300, 500, and 1000 ng/ml VEGF significantly inhibited the phagocytosis of apoptotic cells, with the greatest inhibition caused by 500 ng/ml. The results of treating AECs with 500 ng/ml VEGF for 3, 6, and 9 h are shown in (Figure 1(d)). As the strongest inhibition of phagocytosis occurred after 6 h, epithelial cell cultures were treated with 500 ng/ml VEGF for 6 h in subsequent procedures.

### 3.2. VEGF Inhibition of Phagocytosis Was Not Associated with PS Masking on the Apoptotic Cell Surface

**Figure 2:**
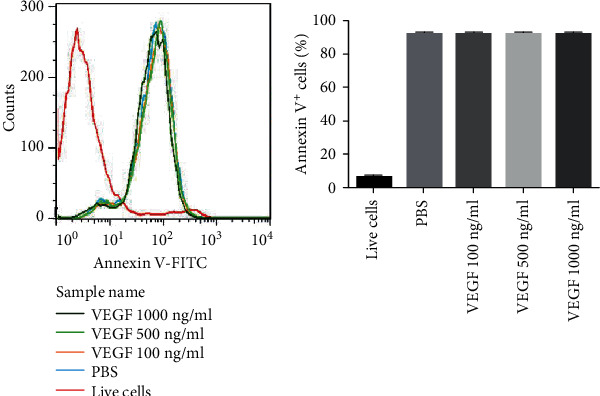
VEGF inhibition of phagocytosis was not associated with masking of PS on the apoptotic cell surface. AECs were inoculated into 12-well plates (5 × 10^4^ cells/well) and treated with curcumin for 72 h before incubation with PBS or 100 ng/ml, 500, or 1000 ng/ml VEGF for 6 h. After washing with PBS, the cell surface PS was assayed by flow cytometry. A representative flow cytometry assay is shown on the left and the figure on the right shows the assay results.

Phagocytosis is mediated by binding PS on the surface of apoptotic cells [12]. Whether VEGF inhibition of phagocytosis associated with PS was investigated by flow cytometry of PS on apoptotic cells after treatment of epithelial cell cultures with VEGF. Compared with PBS controls, there were no significant changes in PS expression by apoptotic cells stimulated by VEGF (Figure 2). The inhibition of the engulfment of apoptotic cells by AECs was not associated with masking PS on the surface of apoptotic cells.

### 3.3. VEGF Inhibition of Phagocytosis Is Mediated by VEGFR2

**Figure 3:**
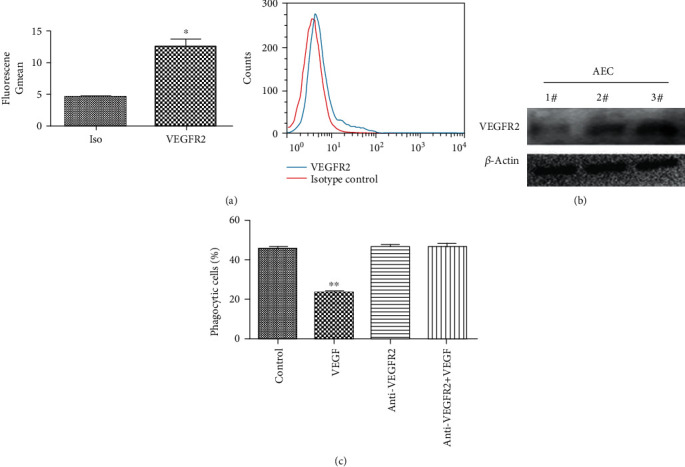
VEGF inhibition of phagocytosis is mediated by VEGFR2. (a) VEGFR2 expression on the surface of AECs was assayed by flow cytometry. A graph of the geometric mean VEGFR2 fluorescence intensity is on the left. ^∗^*P* < 0.05, compared with the Iso group. A representative flow cytometry plot is shown on the right. (b) Western blot assays of VEGFR2 protein expression in three representative epithelial cell lysates (#1-3). (c) AECs were inoculated with 12-well plates (5 × 10^4^ cells/well). After 2 h treatment with anti-VEGFR2 blocking antibody (500 ng/ml), cells were treated with 500 ng/ml VEGF for 6 hours, before incubation with FITC-labeled apoptotic cells for 4 h. Phagocytosis of apoptotic cells by AECs was assayed by flow cytometry. ^∗∗^*P* < 0.01 compared with controls.

VEGFR2 is known to transduce VEGF signaling [13]. As shown in (Figure 3(a)), flow cytometry revealed that VEGFR2 was expressed on the surface of the resting AECs (mean fluorescence intensity = 40.3 ± 5.7 for VEGFR2 versus 7.8 ± 3.6 for the isotype control). VEGFR2 expression was also assayed by western blotting in three airway epithelial cell isolates (Figure 3(b)). Exposure of airway epithelial cell cultures to a VEGFR2 blocking antibody before being VEGF treatment resulted in complete loss of VEGF inhibition of phagocytosis of apoptotic cells (Figure 3(c)). VEGF inhibition of phagocytosis required VEGFR2.

### 3.4. VEGF Inhibition of Phagocytosis Is Mediated by PI3K-Akt Signaling

**Figure 4:**
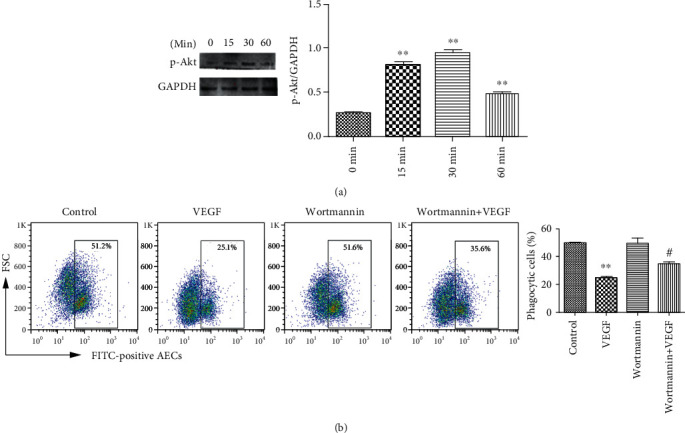
VEGF inhibition AECs phagocytosis is partially mediated by PI3K-Akt signaling. (a) AECs were treated with 500 ng/ml VEGF and phosphorylated Akt expression was assayed by immunoblotting. The left panel is a representative immunoblot, and the results are shown in the graph on the right. ^∗∗^*P* < 0.01 compared with 0 h. (b) AECs were seeded in 12-well plates (5 × 10^4^ cells/well), treated with 1 *μ*M Wortmannin for 2 h, treated with 500 ng/ml VEGF for 6 h, and then incubated with FITC-labeled apoptotic cells for 4 h. Engulfment of apoptotic cells was assayed by flow cytometry. ^∗∗^*P* < 0.01 compared with controls, ^#^*P* < 0.05, compared with VEGF.

PI3K-Akt signaling is known to be stimulated by binding to VEGFR2 [14]. As shown in (Figure 4(a)), VEGF activated Akt as early as 15 min after stimulation. Signaling continued for 45 min and decreased by 60 min. Blocking PI3K-Akt signaling by 1 *μ*M Wortmannin partially blocked the inhibition of phagocytosis by VEGF (Figure 4(b)). This result indicated that the inhibition of phagocytosis by VEGF was partially mediated by PI3K-Akt signaling.

### 3.5. VEGF Inhibition of Phagocytosis Is Associated with Polymerization of Cytoplasmic Actin

**Figure 5:**
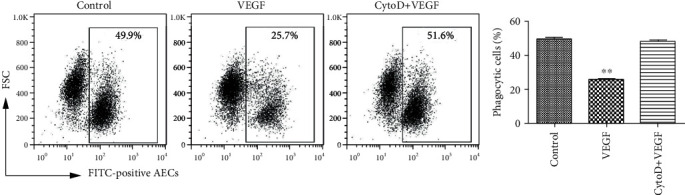
VEGF inhibition of phagocytosis requires polymerization of cytoplasmic actin. AECs were inoculated into 12-well plates (5 × 10^4^ cells/well), pretreated with 200 ng/ml cytochalasin D for 2 h, treated with 500 ng/ml VEGF for 6 h, and then coincubated with FITC-labeled apoptotic cells for 4 h. Phagocytosis was assayed by flow cytometry. The left panel is a representative flow cytometry plot. The right panel is a graph of the result. ^∗∗^*P* < 0.01 compared with controls.

Phagocytosis requires polymerization and depolymerization of cytoplasmic actin regardless of the type of phagocytic receptor or the particle size [15]. To investigate the role of actin in VEGF inhibition of phagocytosis, AECs were pretreated with cytochalasin D, an inhibitor of actin polymerization, before VEGF treatment. Following cytochalasin pretreatment, the percentages of phagocytized apoptotic cells in the VEGF-treated and control cultures were similar (Figure 5). The inhibition of phagocytosis by VEGF involved polymerization of cytoplasmic actin.

### 3.6. VEGF Inhibits Beclin1 Protein Expression in AECs

**Figure 6:**
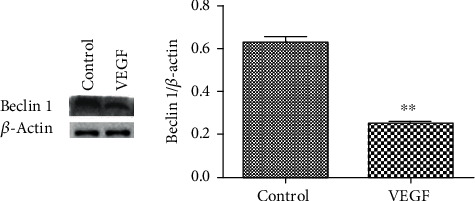
VEGF inhibits Beclin1 protein expression in AECs. AECs were inoculated into 12-well plates (5 × 10^4^ cells/well), and treated with 500 ng/ml VEGF for 6 h before assay of Beclin1 protein expression by immunoblotting. The left panel shows a representative immunoblot; the right panel is graph of the integrated optical density results. ^∗∗^*P* < 0.01 compared with the control.

Beclin1 can bind to phagosomes and phagocyte-associated receptors in the absence of autophagosomes and is involved in macrophage phagocytosis [16]. We investigated whether VEGF affected Beclin1 protein expression in this study. VEGF treatment inhibited Beclin1 protein expression in AECs (Figure 6). This suggests that Beclin1 may also be involved in the phagocytosis by nonprofessional phagocytic cells.

## 4. Discussion

In mammals, the VEGF proteins family members, VEGF-A, -B, -C, -D, and placental growth factor (PLGF), have a characteristic receptor binding pattern that includes endothelial tyrosine kinases including VEGFR1, R2, and R3. VEGF stimulates angiogenesis and lymphangiogenesis mediated by VEGFR2 [17, 18]. Peach et al. reported that binding to VEGFR2 promoted endothelial cell proliferation, survival, migration, and vascular permeability [19]. In this study, VEGF inhibited phagocytosis of AECs following VEGFR2 binding. VEGF is known to activate downstream signaling by PI3K-Akt, PKC, FAK, Ras-MAPK, and other pathways [20]. The PI3K-Akt pathway is present in many types of cells and regulates the activity of downstream proteins that mediate cell proliferation, differentiation, adhesion, migration, apoptosis, and other activities and is associated with inflammation, tumorigenesis, and autoimmune diseases [21, 22]. Ma et al. reported that in a lipopolysaccharide-mediated model of airway inflammation, PI3K-Akt signaling mediated MUC5AC expression and the release of interleukin (IL)-6 and IL-1*β* by AECs, and influenced mucus secretion in AECs by regulating the production of reactive oxygen species [23]. PI3K-Akt activation is also associated with phagocytosis. Yeo et al. found that Fc*γ*R-mediated macrophage phagocytosis was dependent on PI3K-Akt pathway signaling [24], and Lv et al. found that it regulated macrophages phagocytosis of *staphylococcus aureus* [25]. In this study, VEGF activated the PI3K-Akt pathway in AECs, and VEGF inhibition of AECs phagocytosis partially disappeared when the PI3K pathway was blocked. The results indicate that in this study, the PI3K pathway partially mediated VEGF activity required for phagocytosis of apoptotic cells.

Rearrangement of the actin cytoskeleton occurs in all types of phagocytosis and is involved in the acquisition of phagocytic targets, pseudopod extension, encapsulation of particles, and fusion of closed vacuoles regardless of the type of phagocytic receptor and the size of the phagocytic particle [26]. Actin polymerization in phagocytosis is Rac-1-dependent. In this study, VEGF inhibition of phagocytosis completely disappeared after pretreatment of AECs cultures with cytochalasin-D, which inhibits actin polymerization. This indicates that VEGF mediated the inhibition of phagocytosis by its effect on actin polymerization.

In addition to exogenous factors, many intracellular regulatory proteins can regulate phagocytosis in health and disease. Beclin1 is an autophagy-related protein that consists of 450 amino acids and regulates the lipid kinase activity of PI3K catalytic unit in the synthesis of phosphatidylinositol triphosphate. Beclin1 is also associated with phagocytosis, and it can bind to phagosome and phagocyte-associated receptors in the absence of autophagosomes [16]. Lucin et al. reported that glial cells isolated from Alzheimer's disease showed significantly reduced beclin 1 protein levels and Beclin1-mediated glial-cell phagocytosis [27]. In this study, VEGF decreased Beclin1 expression in AECs, suggesting that Beclin1 may also be active in nonprofessional phagocytes.

Nonprofessional phagocytes such as epithelial cells can phagocytose apoptotic cells but not microorganisms. For example, after the cessation of breastfeeding, the clearance of apoptotic mammary epithelial cells is mainly by epithelial cells, not macrophages or other inflammatory cells [28]. Epithelial cells are important structural elements in nearly all types of tissues; consequently, phagocytosis has an important role in maintaining tissue homeostasis. For example, Lee et al. found that the numbers of uncleared apoptotic cells in the intestine of mice with enteritis were increased compared with healthy mice. Increasing the phagocytic activity of the intestinal epithelial cells reduced intestinal inflammation [29]. Similarly, Juncadella et al. reported that inhibition of phagocytosis of apoptotic cells in the airway epithelium resulted in allergic airway inflammation [5]. In this study, VEGF inhibited phagocytosis of AECs, thus increasing the chance of the release of inflammatory molecules from apoptotic cells. That may account for VEGF mediation of airway inflammation in asthmatic mice.

## 5. Conclusion

In conclusion, VEGF inhibited phagocytosis by AECs by activating VEGFR2. The effect of VEGF was not associated with masking PS on the surface of apoptotic cells and was partially mediated by the PI3K-Akt pathway. VEGF activity was associated with inhibition of actin polymerization and decreased Beclin1 protein expression in cultured AECs.

## Data Availability

The data used to support the findings of this study are available from the corresponding author upon request.
